# The treatment of acute traumatic aortic injuries with TEVAR: a retrospective analysis of 19 cases in a level-1 trauma center

**DOI:** 10.1007/s00423-025-03613-y

**Published:** 2025-01-18

**Authors:** F. Liese-Landolt, H.-C. Pape, G. N. Jukema

**Affiliations:** Department of Trauma Surgery, University Hospital Zurich, Rämistrasse 100, CH – 8091 Zurich, Switzerland

**Keywords:** TEVAR, Traumatic aorta injury, Endovascular repair, Trauma management, Chest injury

## Abstract

**Introduction:**

Blunt traumatic aortic injury (TAI) is a critical condition and a leading cause of mortality in trauma patients, often resulting from high-speed accidents. Thoracic endovascular aortic repair (TEVAR) has developed into the preferred therapeutic approach due to its minimally invasive nature and promising outcomes. This study evaluates the safety and efficacy of TEVAR for managing TAI over a 10-year period at a Level-1 trauma center.

**Methods:**

A retrospective analysis was conducted on 19 patients with acute TAI treated with TEVAR between 2012 and 2022 at the University Hospital Zurich. Data were collected from digital records and analyzed according to the Fillinger TEVAR reporting framework. Outcomes included technical success, perioperative complications, and long-term graft durability.

**Results:**

The cohort had a mean age of 39 years and included patients with severe polytrauma. Technical success was achieved in 95% of cases, with no intraoperative deaths or need for open surgical conversion. Perioperative complications were minimal, and the reintervention rate was 5.3%. This study evaluates 10 years of clinical experience managing TAI with TEVAR. Long-term follow-up, with a median duration of 29 months, revealed no graft-related complications or secondary interventions. Imaging confirmed sustained graft integrity, and clinical outcomes were favorable.

**Conclusion:**

TEVAR is a reliable and effective treatment for traumatic aortic injuries, offering excellent technical success rates and minimal perioperative and long-term complications. This study highlights TEVAR’s advantages in managing polytrauma patients and its role as a minimally invasive alternative to open surgery. Additionally, the findings emphasize the need for comprehensive long-term follow-up protocols. Future research should aim to address challenges related to device durability and the integration of advanced imaging techniques to further enhance patient outcomes and broaden the applicability of TEVAR in trauma care.

## Introduction

Blunt traumatic aortic injury (TAI) is one of the leading causes of mortality in trauma patients in the Western world [1]. It generally results from a trauma with an acute deceleration, such as a high-speed motor vehicle accident, as well as from a fall from a height. The acute deceleration produces strong shear forces on the aorta, usually at around the aortic isthmus, which continues on to the comparatively flexible aortic arch, which can be torn from the more fixed descending aorta [2]. Therefore, most traumatic aortic injuries are located at the aortic isthmus (60–90%) [3]; however, the lesions can occur in different parts of the aorta, e.g., at the ascending (8–27%) or descending aorta (11–21%) or the aortic branch (8–18%) [2, 4].

A traumatic aortic injury is often fatal, which is also due to multiple concomitant injuries, including trauma to the head, neck and spine, blunt abdominal trauma, and multiple fractures. Only 9 to 20% of trauma patients with this injury reach the emergency department alive [2, 5]. Trauma to the aorta affects all the layers (intima, media, and adventitia) of the aortic wall differently, from a hematoma in the subintima to a complete aortic rupture. The outcome significantly depends on the adventitia’s integrity and the hematoma formation with its tamponing impact. Patients’ death at the scene of the accident is common, when the TAI reaches the adventitia [1].

In theory, a traumatic injury to the aorta contains a lesion or rupture to the intimal or medial layer. Untreated, after an uncertain amount of time, a rupture of the adventitia (the external aortic wall) occurs [6].

According to the practice guidelines of the SVS (Society of Vascular Surgery) for traumatic aortic injury management, the grading for this injury is based on CT-findings that are assigned to 4 stages: Grade I (intimal tear), Grade II (intramural hematoma), Grade III (pseudoaneurysm) and Grade IV (rupture of the aorta) [7].

Therapeutic options are based on the grade. Injuries with grade I are usually treated with no intervention, due to their tendency to stop bleeding on their own. Grade II is in a “gray zone” and can be managed either with an intervention or conservatively with no immediate intervention. For grades III and IV, an intervention is the current standard of care [7, 8]. Proper treatment and time management is indispensable when facing the often multiple and complex injuries [9].

The use of thoracic stent grafts was initially planned to treat aneurysmal diseases. Dake et al. published first results of the successful use of a stent graft for thoracic aneurysm of the descending aorta in highly selected patients in 1994. Semba et al. showed in 1997 that endovascular treatments with a stent graft are technically possible in an acute descending aortic rupture. Thoracic endovascular aortic repair (TEVAR) has revolutionized the therapy of TAI over the last 20 years [7].

With a less invasive character that reduces perioperative morbidity and mortality, TEVAR recently became the gold standard for TAI [3, 9, 10]. The study goal is to provide an overview of TEVAR over the last 10 years at the University Hospital Zurich that includes 19 patients with TAI who underwent TEVAR intervention. In 2010, Fillinger and colleagues proposed a standardized reporting system for TEVAR intervention and outcomes, emphasizing clear, comparable endpoints such as technical success, perioperative complications, and long-term graft durability. Adoption of such frameworks has enhanced the consistency and clinical relevance of TEVAR research [11]. This study seeks to assess the effectiveness and safety of TEVAR for managing TAI at a Level-1 trauma center over a 10-year period, utilizing the TEVAR reporting system to structure and interpret outcomes.

## Patients and methods

This retrospective, observational cohort study was conducted to evaluate the clinical result of thoracic endovascular aortic repair (TEVAR) in managing traumatic aortic injuries (TAIs) using the standardized reporting framework proposed by Fillinger et al. (2010) [11]. Data were collected and analyzed according to these endpoints.

### Patients

We retrospectively analyzed 19 patients with traumatic thoracic aortic injuries treated with TEVAR at the University Hospital Zurich, a Level-1 trauma center, between 2012 and 2022. Inclusion criteria were confirmation of acute traumatic aortic injuries diagnosed through CTA, managed using TEVAR, while patients undergoing conservative treatment or open surgical repair were excluded. All patients were treated following the Advanced Trauma Life Support (ATLS) protocol.

### Methods

Ethical approval for this study was obtained from the institutional ethics board (EKNZ 2018–01936). Data were collected from digital patient records, including demographic details, clinical presentation, imaging findings, procedural details, and follow-up outcome.

Patients presenting with life-threatening injuries underwent immediate stabilization; these included chests drain insertion, laparotomy for abdominal hemorrhage or trauma, and/or therapy of an intracranial hemorrhage. TEVAR was performed after stabilization, with a median time from injury to procedure of two days (range: 2 h to 12 days). Preoperative imaging was conducted using spiral computed tomography angiography (CTA) with intravenous contrast.

Endpoints were categorized as follows:

- Technical Success (Accurate deployment of the endograft, absence of intraoperative complications, including endoleaks.)
- Perioperative Complications (Occurrence of adverse events within 30 days of the procedure, such as graft-related issues, thromboembolic events, or infections.)
- Long-term Outcomes (Rates of reintervention, graft migration, or occlusion, Long-term survival and absence of graft-related complications.)

### Procedural details

All procedures were performed by a vascular surgeon teamed up with an interventional radiologist in an endovascular operating room with the possibility to convert to open surgery.

Two main types of self-expanding, market-available endografts were used: Gore-TAG (WL Gore and Associates, Flagstaff, Arizona), TX2 (Cook Medical Inc., Bloomington, Indiana).

Patients were placed in a supine position, and femoral artery access was achieved under ultrasound guidance. In one case, both femoral and brachial access were utilized. A heparin bolus was administered intraoperatively, except in cases with contraindications such as polytrauma and bleeding risk. Perioperative antibiotic prophylaxis with intravenous cefuroxime was administered. In all patients, a minimum of 2 Perclose ProGlide devices (ABBOTT Vascular INC., Redwood City, California) were used. To ensure angiographic control throughout the intervention, a pigtail catheter was placed. Fluoroscopic guidance was used to ensure proper positioning of the endograft. According to prior imaging, the endograft was deployed. A final angiogram was performed to rule out any endoleak and to confirm the correct positioning of the graft. The surgical techniques and deployment methods have been thoroughly detailed in previous reports [12, 13].

### Postoperative care and follow-up

Postoperatively, patients underwent CTA within the first few days to confirm graft positioning and exclude complications such as endoleaks. Antiplatelet therapy with aspirin 100 mg daily was initiated in all patients. Follow-up imaging, including CTA or magnetic resonance imaging (MRI) for younger patients, was scheduled at 3–6 months, followed by annual assessments thereafter as recommended [14]. After 36 months without abnormalities in imaging, the CTA Follow-up is every two to five years [14].

Technical success was defined as successful placement and deployment of the TEVAR at the target location, without the need to convert to open repair, death within 24 h or other intraoperative complications, including endoleaks type I-III on intraoperative completion angiography [11]. An endoleak was clarified as blood outflow into the lesion despite the placed stent graft.

### Statistical analysis

Descriptive statistics were applied to summarize the characteristics, procedural aspects and clinical outcomes. Data were presented as mean ± standard deviation or median with interquartile range, categorical variables are expressed as n in %, and analyzed using Microsoft Excel.

## Case report

In addition to the cohort analysis, a detailed case report was included to illustrate the application of TEVAR in a unique and complex trauma scenario, emphasizing specific challenges and outcomes not captured by the broader cohort.

**Image 1:**
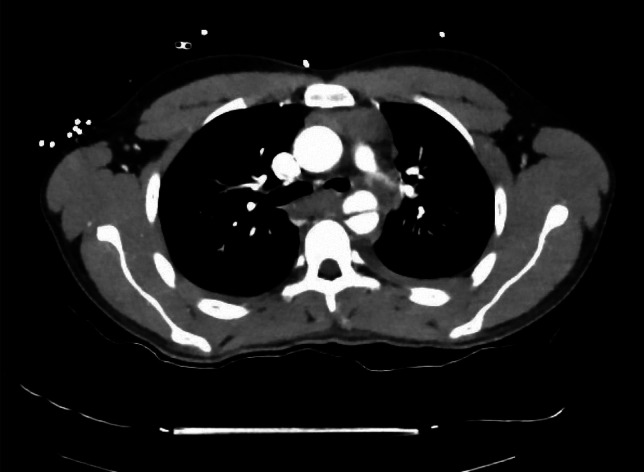
Preoperative CT-imaging

A 17-year-old male patient was transferred to the emergency room at our Institution after a high-speed car accident as a passenger. The patient presented a Grade III traumatic aortic injury at the isthmus affecting the LCA, hemopneumothorax, fractures of the ribs, pelvis, and femur, as well as injuries to the spleen, liver, and right kidney, resulting in a polytrauma with an ISS of 57. As described, the initial trauma management followed the ATLS protocol. Acute life-threatening hemopneumothorax, was treated with chest tube insertion. Preoperative imaging using CTA with iv. contrast enhancement confirmed the Grade III aortic injury (shown in Image 1).

**Image 2:**
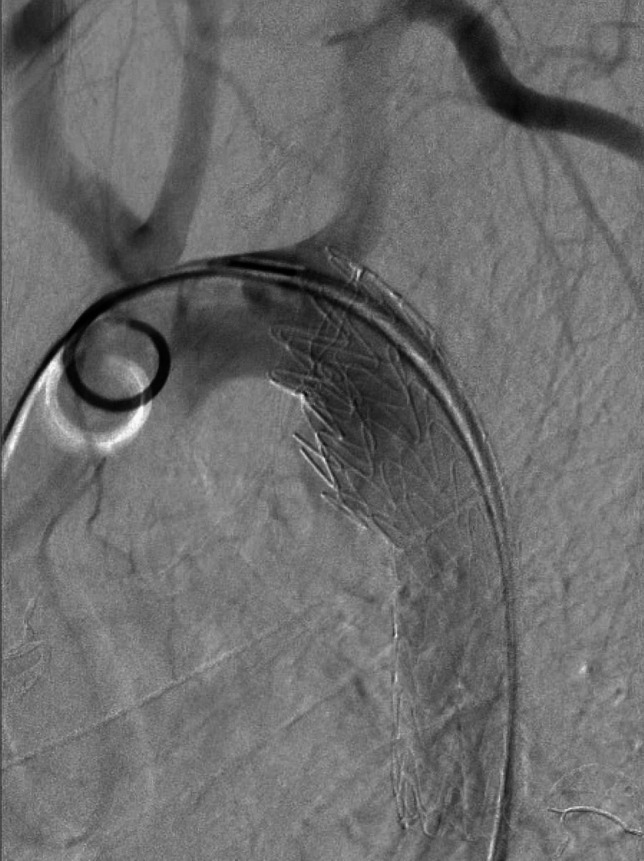
Intraoperative fluoroscopy during graft placement

Within five hours of the accident, a TEVAR was performed in an endovascular operating room equipped for conversion to open surgery if necessary. A Gore TAG endograft with a diameter of 28 mm and a length of 100 mm was deployed under fluoroscopic guidance, with intraoperative angiography ensuring proper positioning and exclusion of endoleaks (Image 2). The operation lasted 45 min and was conducted under general anesthesia. Standard prophylactic antibiotic therapy with intravenous Cefuroxime and a heparin bolus were administered. The fractures of the pelvis and femur were stabilized with an external fixator postinterventionally and definitively treated in a subsequent operation. Postoperatively, the patient was monitored in the Intensive Care Unit (ICU) for nine days and hospitalized for a total of 17 days, making a quick and pleasant recovery. Postoperative CTA revealed no complications related to the endograft. Aspirin 100 mg daily was prescribed to reduce the risk of graft occlusion.

**Image 3:**
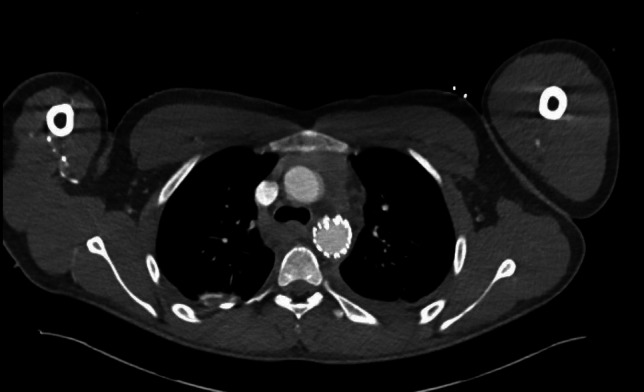
Postoperative CT-imaging after implantation of a thoracic stent

**Image 4:**
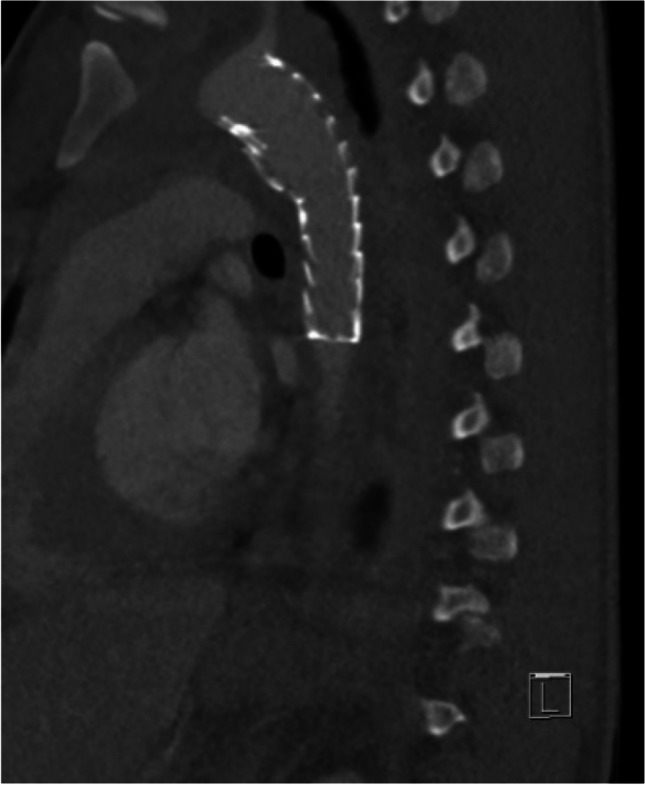
Postoperative CT-imaging after implantation of a thoracic stent

The patient was followed up for three years, during which he reported no thoracic complaints or complications related to the TEVAR. Imaging follow-up, performed with a CTA, confirmed the graft’s stability and functionality without signs of migration, endoleak, or infection (Images 3 and 4). This case exemplifies the successful application of TEVAR, adhering to the protocols outlined in our study, in managing complex polytrauma with traumatic aortic injury.

## Results

We successfully treated 19 patients who suffered from TAI treated with an endovascular graft. Of those, 17 patients suffered a high velocity accident with a sudden deceleration (10 car accidents, 1 motorcycle accident, and 5 falls from heights) and 2 were run over by a car. All of them had multiple and complex injuries, as shown in Table 1, and were treated with TEVAR after assessment and initial stabilization.

**Table 1:** Concomitant injury

Concomitant injury	*n*	%
Head Injury		
Traumatic brain injury	14	73.6
Brain bleeding	4	21
Skull/facial fracture	4	21
Paraplegia	2	10.5
Abdominal injury		
Diaphragm rupture	1	5.2
Spleen	7	36.8
Pancreas	2	10.5
Renal and adrenal	4	21
Intestine	1	5.2
Liver	5	26.3
Cardiopulmonary injury		
Pericard rupture	1	5.2
Contusion cordis	5	26.3
Pulmoary contusion	8	42.1
Hemato or pneumothorax	11	57.9
Fractures		
Sternal	8	42,1
Clavicula	4	21
Rib	10	52.6
Scapula	1	5.2
Spine	11	57.9
Sacrum	5	26.3
Pelvic	8	42.1
Extremities	9	47.3

As outlined in the patient’s demographic and aortic injury characteristics are summarized in Table 2. The mean age was 39 years (range 17–85 years). Among the patients, 63% were male, and the median GCS upon arrival at the emergency department was 12, with a range from 3 to 15. 94.7% presented with an American Society of Anesthesiologists score (ASA Score) of 3 or more. The Injury Severity Score (ISS) was assessed for each study participant, with a mean of 34.2, ranging from 17 to 66.

**Table 2:** Patients characteristics

	*n*	%	Mean
Age, y			39.4 (17–85)
Male	12	63.1	
Female	7	36.8	
GCS			11.9 (3–15)
ISS			34.2 (17–57)
ASA ≥ 3	18	94.7	
Car accident	10	52.6	
Motorcycle accident	1	5.2	
Fall from height	5	26.3	
Auto vs. pedestrian	2	10.5	
Type of thoracic injury			
Grade I	2	10.5	
Grade II	11	57.9	
Grade III	4	21	
Grade IV	2	10.5	
Site of lesion			
Isthmus affecting LCA	5	26.3	
Isthmus not affecting LCA	8	42.1	
Aorta descendens	3	15.7	
Aorta abdominalis	2	10.5	
Time to intervention, h			44.6 (2–308)

Most patients had a pseudoaneurysm at isthmus level; only 4 patients had a rupture of the aortic wall. Only 3 patients had a TAI at the level of the aorta descendens. Two patients were included with a traumatic aorta injury of the aorta abdominalis also treated with an endovascular endograft. None had an injury at the aorta ascendens level.

The traumatic aorta injury was primarily suggested due to a hemato- or pneumothorax, a mediastinal hematoma or a contour abnormality, detected in a thoracic xray, as shown in 68.4% of the patients. Furthermore, according to the ATLS guidelines, the TAI was confirmed in all patients by a computed tomographic angiography (CTA).

The mean time from injury to the endovascular procedure was 44 h, with a range from 2 h to 12 days. Of the patients, 15 were treated within the first 24 h. In three patients, a significant delay was noted. The reasons were delayed diagnosis of the injury (1 patient), initially attempt for conservative treatment (1 patient), and multiple previous emergency surgeries (1 patient).

Gore-TAG (WL Gore and Associates, Flagstaff, Arizona) was used in 16 cases, while Zenith TX2 (Cook Medical Inc., Bloomington, Indiana) in three patients. The sizing of the endovascular grafts that were used was determined in a pre-operative CTA measurement. In one patient, 2 Gore-Tags were used because of a long-distance dissection. The median width of the stent graft was 30mm (IQR, 21-37mm) with a median length of 100mm (IQR, 80-150mm). In 5 patients, the TAI was localized directly near the origin of the left subclavian artery.

In 3 patients, an intentional overstenting of the left subclavian artery (LSA) was performed. None of these patients experienced a left arm or verterobasilar ischemia, so revascularization of the LSA was not needed.

In a different patient, 2 Gore Viabahn endoprostheses (WL Gore and Associates, Flagstaff, Arizona) were used to ensure blood flow to the LSA. The periprocedural details are shown in Table 3.

**Table 3:** Periprocedural details

	*n*	%	Mean
General anesthesia	13	68.4	
Locoregional anesthesia	6	31.6	
Periprocedural Data			
Time of Intervention—min			70 (20–210)
Fluoroscopy—min			9:24 (3–14)
Arterial Access Problems	0	0	
Conversion to open Repair	0	0	
Endoleak	2	10.5	
Technical data			
TX2	3	15.6	
Gore-TAG	16	84.3	
Diameter of graft (mm)			30.37 (23–40)
length of graft (mm)			106.3 (80–150)
Coverage of the LSA	3	15.7	
Post Intervention			
In hospital mortality	1	5.2	
Survival	18	94.7	
Ventilator days			9.1 (0–90)
ICU days			8.9 (1–24)
Hospital days			25 (5–98)
Renal failure	2	10.4	
ARDS	2	10.4	
Pneumonie	5	26	

Mean operating time (skin-to-skin) was 70 min (20–210 min) with an fluoroscopy time of 9 min and 24 s (3–14 min).

According to prior imaging the endograft was deployed correctly, in just 1 case, ballooning had to be used at both ends of the endograft to adjust and obtain proper fixation.

The technical success rate of TEVAR in this cohort was 95%. Accurate graft deployment was achieved in all 19 patients. Intraoperative angiograms confirmed proper graft positioning and the absence of significant endoleaks in 18 cases. One patient experienced an intraoperative Type II endoleak, which resolved spontaneously during follow-up imaging. No conversions to open surgery were required, and no intraoperative deaths occurred.

### Perioperative complications

Perioperative complications within 30 days were minimal.

Only 1 patient died postoperatively in the ICU due to a hypoxic ischemic encephalopathy with multi-organ failure due to a pulmonary embolism after vertebroplasty of the thoracic and lumbar spine.

All patients underwent a postinterventional CTA. In 1 patient, a hypoperfusion of the left carotid artery and an Endoleak Type Ia was detected. He underwent a second intervention with implantation of a carotid stent.

With the exception of one patient, all received anti-thromboembolic therapy with heparin. Nevertheless, 1 patient suffered from a leg ischemia triggered by an acute occlusion of the external iliac artery and an occluding thromboembolism of the truncus tibiofibularis. A revision intervention with a thrombectomy and a stenting of the external iliac artery was performed. This patient suffered of no consequential damage after the second intervention.

The mean hospitalization was 25 days (range, 5–98 days) with a mean intensive care unit time of 8.89 days (range 1 to 24 days). One patient had a major traumatic brain injury with multiple cerebral hemorrhage and impaired cognitive outcome. For this reason, after 24 days in the ICU, he was transferred to a neurological facility close to his hometown.

### Long-term outcomes

A chest CTA was performed in every patient before discharge. Clinical evaluations and imaging assessments were conducted at 3, 6, and 12 months, with data available for all patients. In two patients imaging was performed with MRI due to young age. Follow-up was conducted for a median duration of 29 months (range: 4–112 months). There was one late mortality unrelated to the TAI or TEVAR procedure. No graft-related complications, such as migration, occlusion, or infection, were observed during the follow-up period. One patient presented with an asymptomatic occlusion of the common carotid artery, requiring no intervention. Reintervention rates were 0% beyond the perioperative period during follow-up.

Imaging follow-ups demonstrated sustained graft integrity with no instances of secondary endoleaks or structural compromise. Patients with overstenting of the left subclavian artery (*n* = 3) experienced no ischemic complications, and collateral circulation appeared sufficient, confirmed by imaging, without ischemic complications. Clinical outcomes were favorable, with no significant thoracic complaints reported during the follow-up visits.

### Summary of key metrics

One intraoperative complication (Endoleak Type II), with a Perioperative Mortality 5.3% (1/19 patients) and a Reintervention Rate 5.3% (1/19 patients, within 30 days). The Long-term Graft Durability was 100%.

This analysis underscores the efficacy and safety of TEVAR in managing traumatic aortic injuries, with excellent technical success rates and minimal perioperative and long-term complications. The findings align with the structured endpoints outlined in the Fillinger framework, supporting the utility of standardized reporting in TEVAR studies.

## Discussion

### Advantages of TEVAR

TEVAR offers several advantages over traditional open repair, particularly for polytrauma patients [15]. Its minimally invasive nature allows for stabilization of other life-threatening injuries before addressing the aortic injury, reducing perioperative risks. Additionally, TEVAR eliminates the need for thoracotomy and aortic cross-clamping, which are associated with significant risks of respiratory complications and spinal cord ischemia [1, 16]. In this study, delayed interventions in three patients due to stabilization requirements and change in treatment further demonstrate TEVAR’s flexibility and adaptability to complex clinical scenarios.

Since the first endovascular treatment was performed to treat TAI in 1997, according to guidelines from the European society for Vascular Surgery (ESVS) and the SVS, TEVAR has become the gold standard treatment for the thoracic aorta in patients with suitable anatomy, supported by prior evidence demonstrating its superiority over open surgery [14, 16–18].

TEVAR allows a minimally invasive therapeutic approach to treating critically ill patients who may have not survived an open repair. It also allows the delay of the intervention after treating acute life-threatening injuries in order to stabilize the patient before they undergo the procedure [4].

The case report in this Paper demonstrated a favorable outcome consistent with the broader cohort, despite additional challenges posed by polytrauma. This underscores TEVAR’s flexibility in managing high-risk scenarios while aligning with established endpoints.

### Comparison with existing literature

This study demonstrates the efficacy and safety of (TEVAR) in managing blunt traumatic aortic injuries. Our findings show a 95% technical success rate, minimal perioperative complications, and excellent long-term graft durability over a median follow-up of 29 months. The 95% technical success rate in this study aligns with prior reports emphasizing the precision and reliability of TEVAR in traumatic settings [3, 19]. Previous studies have reported technical success rates ranging from 95 to 100% in similar patient populations, highlighting the procedure's feasibility even in complex trauma scenarios. As is shown, no death due to the intervention and no aorta-related deaths were observed during Follow-up, supporting the promising long-term outcomes of endovascular repair for aortic traumatic injury [20].

Lower-grade injuries, such as Grade 1 and Grade 2, are less commonly managed with TEVAR, consistent with their rarity and the efficacy of conservative management for minimal intimal injuries [3]. The comparison reveals that a greater number of patients with Grade II (57.9%) TAI underwent surgery than in comparable studies (14–20%) [3]. Grade II patients are situated in a grey zone, where a careful decision must be made between intervention and conservative management. Patients with non-stable conditions with a high ISS are more likely to undergo an Intervention even for Grade II injuries. In these situations, clinical expertise and decision-making are critical to ensuring the best possible outcome [3, 21].

Perioperative complications in this cohort were minimal, with a perioperative mortality rate of 5.3%, which is lower than reported rates for open surgical repair, often exceeding 10% [3]. The absence of paraplegia or major ischemic complications in our patients underscores the advantages of TEVAR’s minimally invasive approach [17].

In this study, the mean operating time is just over 1 h (1 h, 10 min), which is comparable to the operating time reported in the literature [18]. The mean ICU stay (9 days) and the mean hospital days (25 days) are highly dependent on the concomitant injuries, but were overall shorter than those of patients with an open surgery repair [22] and align with similar studies.

The timing of intervention for blunt thoracic aortic injuries (BTAI) is crucial in achieving optimal patient outcomes. Current guidelines recommend repair within 24 h if significant concurrent injuries have been treated or stabilized [14]. However, the severity of the injury must also guide timing, as Grade III injuries, with ruptured intima and media but intact adventitia, allow for delayed repair for 24 h due to temporary containment by the adventitia [3]. In our study, the mean time from injury to TEVAR was 44 h, with 15 patients treated within the first 24 h. Three patients experienced delays due to factors such as stabilization of concomitant injuries, delayed diagnosis, or initial attempts at conservative management, yet no adverse outcomes were observed in these cases. These findings align with Alarhayem et al., who demonstrated a survival benefit for patients undergoing delayed TEVAR beyond 24 h when injury severity was comparable, and no signs of imminent rupture were present [23]. Their cohort of 2821 patients found mortality benefits in delayed repair, irrespective of injury severity [23]. This evidence supports the importance of individualized timing strategies, balancing the urgency of repair with the need to address other life-threatening injuries, as reflected in the favorable outcomes of our delayed intervention cases.

Despite its advantages, TEVAR has its technical difficulties. In our study, the left subclavian artery was deliberately covered in three patients throughout the endovascular repair. Although none of the patients had complications and tolerated the overstenting reasonably well without any upper extremity claudication and fatigue on exertion, which had rarely been described in the literature after overstenting. A left-sided mammary bypass and a dominant left vertebral artery are a clear contraindication for overstenting the LSA, which was not present in our patients. A preoperative CTA is mandatory to display the contralateral vertebral artery and preoperative planning [2, 24].

An increased risk of endoleakage has been described with an incomplete endograft positioning or the oversizing of the endograft [25]. In this study, 2 patients showed an endoleak. One patient with a post-operative endoleak Type Ia underwent an immediate re-intervention. Another patient with a type II endoleak was closely monitored until the endoleak resolved on its own. To date, no adverse event has been detected in our cohort.

A further point of concern is the long-term follow-up and the subsequent doses of radiation due to the CTA imaging. The patient population with traumatic aorta injuries is mostly young in age with a considerable life expectancy that exceeds the current experience with TEVAR [1, 2, 16]. Patients who have been treated with an endovascular graft after traumatic aorta injury are looking forward to a long and closely monitored follow-up period [16]. Therefore, we established a follow-up with magnetic resonance imaging in very young patients to minimize the radiation exposure. Special follow-up protocols may need refinement.

### Limitations

Despite its strengths, this study has limitations. The small sample size and single-center design restrict generalizability. The retrospective nature introduces potential biases, such as selection bias and incomplete data capture. Additionally, while follow-up was comprehensive, longer follow-up periods beyond 29 months are desirable and necessary to assess device durability and long-term patient outcomes beyond the observed 10 years.

### Future directions

Future research should focus on multi-center, prospective studies with larger cohorts to validate these findings. Long-term studies are needed to refine follow-up protocols, address radiation exposure concerns from repeated imaging, and evaluate device durability in younger patients with longer life expectancies. Additionally, the role of alternative graft designs and evolving imaging technologies in improving outcomes warrants further investigation.

### Conclusions

The findings of this study indicate that endovascular intervention is a reliable and efficient treatment option for patients suffering from TAI, with minimal mid-term morbidity and mortality rates, as seen after 10 years of clinical observation. The follow-up of these 19 patients who underwent TEVAR for acute aortic injury in our institution is encouraging. Endovascular therapy is gold standard for patients with polytrauma and TAI, but we must not forget the young age of most of the patients, as the thoracic aorta naturally enlarges with age, extended follow-up is essential to monitor potential mismatches between the endograft and the aorta [18, 26]. While our findings are promising, further research is essential to optimize treatment strategies, refine follow-up protocols, and evaluate long-term outcomes in larger patient populations.
